# Antipathogenic Activity of Betainized Polyethyleneimine Sprays Without Toxicity

**DOI:** 10.3390/biomedicines12112462

**Published:** 2024-10-26

**Authors:** Selin S. Suner, Ramesh S. Ayyala, Nurettin Sahiner

**Affiliations:** 1 Department of Chemistry, Faculty of Sciences, Canakkale Onsekiz Mart University Terzioglu Campus, Canakkale 17100, Turkey; sagbasselin@gmail.com; 2 Department of Ophthalmology, Morsani College of Medicine, University of South Florida, Tampa, FL 33612, USA; rayyala@usf.edu; 3 Department of Bioengineering, U.A. Whitaker College of Engineering, Florida Gulf Coast University, Fort Myers, FL 33965, USA

**Keywords:** polyethyleneimine, betainized polyethyleneimine, antipathogenic sprays, biocompatible, antimicrobial, antiviral

## Abstract

**Background/Objectives**: The design of alternative antipathogenic sprays has recently attracted much attention due to the limitations of existing formulations, such as toxicity and low and narrow efficacy. Polyethyleneimine (PEI) is a great antimicrobial polymer against a wide range of pathogens, but toxicity limits its use. Here, betainized PEI (B-PEI) was synthesized to decrease the toxicity of PEI and protonated with citric acid (CA), boric acid (BA), and HCl to improve antimicrobial activity. **Methods**: Cytotoxicity of the PEI-based solutions was determined on L929 fibroblast cells. Antibacterial/fungal activity of PEI-based antipathogenic sprays was investigated by microtiter and disc diffusion assays, in addition to bacterial viability and adhesion % of common bacteria and fungi on the PEI-treated masks. Furthermore, the antiviral effect of the PEI-based solutions was determined against SARS-CoV-2 virus. **Results**: The biosafe concentration of PEI was determined as 1 μg/mL with 75 ± 11% cell viability, but B-PEI and its protonated forms had great biocompatibility even at 1000 μg/mL with more than 85% viability. The antibacterial/fungal effect of non-toxic B-PEI was improved by protonation with BA and HCl with 2.5–10 mg/mL minimum bactericidal/fungicidal concentrations (MBCs/MFCs). Bacterial/fungal viability and adhesion on the mask was almost eliminated by using 50 μL with 5–10 mg/mL of B-PEI-BA. Both protonated bare and betainized PEI show potent antiviral activity against SARS-CoV-2 virus. **Conclusions**: The toxicity of PEI was overcome by using betainized forms of PEI (B-PEI). Furthermore, the antimicrobial and antiviral efficacy of PEI and B-PEI was improved by protonation with CA, BA, and HCl of amine groups on B-PEI. B-PEI-BA spray solution has great potential as an antipathogenic spray with broad-spectrum antimicrobial potency against harmful bacteria, fungi, and viruses without any toxicity.

## 1. Introduction

In recent years, bacterial, fungal, or viral infections have remained the cause of major problematic diseases worldwide [1]. These pathogenic microorganisms, especially causing hospital-acquired infections, are spreading rapidly and leading to nearly 10 million deaths per year, which comprise of 15% of total fatalities [2]. Treatment of infections places an economic burden on healthcare systems due to costly antibiotic treatment, long-term hospitalization, time, etc. [3]. Antipathogenic solutions have great potential to eliminate or eradicate harmful microorganisms and could be used for various purposes such as microbial elimination, sterilization of public places, as alternatives to agricultural pesticides [4], preventing bactericidal resistance [5], and inhibiting the spread of infections from contaminated surfaces [6]. Alcohols, quaternary ammonium salts, hydrogen peroxide, sodium hypochlorite, phenolic compounds, povidone-iodine, chlorhexidine [7], aldehydes, organic acids, metal derivatives [8,9], metal nanoparticles [1], surfactants, and some essential oils and plant extracts [4] are the common ingredients in antipathogenic spray formulations. However, toxicity to humans via inhalation or skin absorption, harmful effects for the environment, and low or specific antipathogenic activity are the major problem with their common use [10]. There is an urgent need for and growing interest in the design and development of novel antipathogenic sprays to prevent microbial colonization and dispersal.

Branched polyethyleneimine (PEI) is a well-known cationic polymer composed of primary, secondary, and tertiary amine groups at a 1:2:1 ratio [11]. PEI is generally used in a wide range of industrial applications as a detergent, adhesion promoter [12], water removal agent [13], and in separation technology [14], as well as for specific biotechnological applications such as gene [15] or DNA therapy [16], tissue regeneration [17], protein interaction [18], antigen bonding, and especially microbial treatments [19]. PEI exhibits a potent antipathogenic effect against broad-spectrum airborne and waterborne microorganisms [20,21]. In fact, PEI can interact with the influenza virus and improve local and systemic immune responses [22]. The microbial inhibition mechanism of PEI was reported to occur in three different ways. One is the electrostatic interaction ability of the more cationic PEI with the negatively charged membrane of microorganisms. This interaction damages the integrity of the membrane structure. In addition, PEI can impair some cellular pathways, such as enzyme activity. The other is the binding effect of PEI to DNA and RNA of the bacteria, fungi, or viruses, which prevents the replication or transcription of the microorganism’s genetic material. Finally, PEI can destroyed proteins, lipids, and DNA by generating reactive oxygen species (ROS) [23]. In addition to its microbicidal activity [24], PEI has a synergistic bacteriostatic effect when combined with a wide range of antibiotics [25]. As reported by Lan et al., PEI is not only a broad-spectrum antibacterial agent but also has a great biofilm eradication ability against antibiotic-resistant bacteria [26]. Similar to the antibacterial effects, PEI stimulates oxidative stress in human cells by ROS generation [27], membrane and mitochondrial disruption [28], and DNA damage, and it has inflammatory effects in humans [16]. Therefore, the toxicity of branched PEI limits its direct use in health products and biological applications [29]. In the last decade, PEI has been commonly used as a antimicrobial coating agent for devices and surfaces [30], for the synthesis of polyelectrolyte complexes [19], generally as a modifying agent for polymeric structures or metal nanoparticles [21], and in direct use in the synthesis of polymeric networks, related to its excellent antimicrobial activity and polycationic nature. Palantoken et al. reported that quaternary ammonium polyethyleneimine-based hydrogels had a great antimicrobial effect against adherent harmful bacteria [31]. In a study, colloid PEI–silver nanoparticles were prepared to improve the antibacterial activity of Ag nanoparticles, but the PEI concentration limited the stability of the colloidal dispersion and was significantly toxic for human fibroblast cells [21]. Ortega et al. investigated the antibacterial activity of quaternary ammonium PEI nanoparticles against clinical pathogenic bacteria. These quaternized PEI-based nanoparticles had excellent antibacterial activity as an alternative to antibiotics for peritonitis treatment, but they could only be used up to a 50 μg/mL concentration because of their toxicity [32].

The objective of this study was to develop antipathogenic spray solutions derived from PEI. The aim was to overcome the toxicity of PEI via the betainization of PEI chains to form B-PEI, which was synthesized in a single step using 1,3-propane sultone as the betainizing agent. To improve the microbicidal activity, PEI and B-PEI were protonated with well-known antiseptic molecules such as citric acid (CA), boric acid (BA), and HCl. The cytotoxicity of the PEI sprays was investigated on L929 fibroblast cells to compare the potential uses of these sprays in terms of toxicity. Antimicrobial effects of the prepared PEI-based solutions against common pathogens including *E. coli* (ATCC 8739), *P. aeruginosa* (ATCC 10145), and *K. pneumoniae* (ATCC 700603) as Gram-negative bacteria, *S. aureus* (ATCC 6538) and *B. subtilis* (ATCC 6633) as Gram-positive bacteria, *C. albicans* (ATCC 10231) and *Mucor* spp. as fungal strains, and SARS-CoV-2 virus were evaluated by standard tests including microtiter broth dilution and disc diffusion assays. Furthermore, the antipathogenic activities of these spray solutions were also determined in regard to their microbial inhibition abilities against the hazardous bacteria and fungi on face masks. In addition, the bacterial and fungal adhesion% on face masks was investigated in the presence of PEI-based spray solutions in comparison to the control group. So that the potential use of PEI and B-PEI formulations for their broad-spectrum antimicrobial activity in antipathogenic sprays was determined.

## 2. Materials and Methods

### 2.1. Materials

Polyethyleneimine (PEI, 50 wt.% aq. solution, branched, M_n_: 60,000, Acros Organics, Waltham, MA, USA), 1,3-propane sultone (98%, Aldrich-Aldrich, Carlsbad, CA, USA), hydrochloric acid (HCl, ACS reagent 37%, Sigma-Aldrich, St. Louis, MO, USA), sodium hydroxide (NaOH, ≥97%, Pellets/Certified ACS, Fisher Chemical, Pittsburgh, PA, USA), boric acid (BA, 99%, ACS reagent, Sigma-Aldrich, USA)), and citric acid (ACS reagent ≥99.5%, Sigma-Aldrich, USA) were purchased and used as received. L929 fibroblast cells (Mouse C3, a connective tissue) were supplied by the SAP institute, Ankara, Turkey. Trypsin (0.25%, EDTA 0.02% in PBS, Sigma-Aldrich, USA), Dulbecco’s Modified Eagle’s Medium (DMEM, with 4.5 g/L glucose, 3.7 g/L sodium pyruvate, L-Glutamine 0.5 g/mL, ThermoFisher Scientific, Waltham, MA, USA), fetal bovine serum (FBS, heat inactivated), and penicillin/streptomycin (10,000 U/mL penicillin, 10 mg/mL streptomycin, ThermoFisher Scientific, Waltham, MA, USA) were obtained from Pan Biontech GmbH, Aidenbach, Germany). Dimethyl sulfoxide (DMSO, 99.9%, Carlo Erba, Val-de-Reuil, France), trypan blue (0.5% solution, Biological Industries, Göttingen, Germany), and 3-(4,5-dimethylthiazol-2-yl)-2,5-diphenyltetrazolium bromide (MTT agent, BioFroxx, Einhausen, Germany) were used as received. *Escherichia coli* (ATCC 8739), *Pseudomonas aeruginosa* (ATCC 10145), and *Klebsiella pneumoniae* (ATTC 700603) as Gram-negative bacteria, *Staphylococcus aureus* (ATCC 6538) and *Bacillus subtilis* (ATCC 6633) as Gram-positive bacteria, and *Candida albicans* (ATCC 10231) as a fungus were purchased from KWIK-STIK, Microbiologics, Chalon-sur-Saône, France. The *Mucor* spp. fungal strain was obtained from the Microbiology Department of Canakkale Onsekiz Mart University, Turkey. Nutrient agar (NA, with 15 g/L bacteriological agar, 5 g/L peptone, and 3 g/L meat extract, Condolab, for cultivation, Madrid, Spain), nutrient broth (NB, with 5 g/L peptone and 3 g/L meat extract, for microbiology, Merck, Darmstadt, Germany), and potato dextrose agar (for microbiology, Merck, Darmstadt, Germany) were used as growth media for microorganisms. Gentamicin sulfate (>590 IU/mg gentamycin, Acro Organics, Belgium, WI, USA) and amphotericin b (AMB, 85%, Acros Organics, USA) were used as antibiotic and antifungal drugs. A cloth mask (100% cotton) was used in antimicrobial studies. All other solvents such as acetone (99+%) and ethanol (98%) were high purity and used as supplied. DI water was obtained from Millipore-Direct Q UV3 at 18.2 MΩ.cm to prepare all aqueous solutions.

### 2.2. Preparation of Betainized PEI

Polyethyleneimine (PEI) was betainized by treating PEI with 1,3-propane sultone (PS) according to the procedure described by Sahiner et al. [33]. In brief, 20 g of PEI at 50 wt% in water was added to 20 mL of 1 M NaOH solution and stirred at 500 rpm for 30 min at room temperature. Then, 41.6 g of 1,3-propane sultone (PS) at a 1:1.5 molar ratio based on PEI repeating units (assuming 44 g/mole) was dissolved in 450 mL of DI water at a 500 rpm stirring rate at room temperature to prepare the PS solution. The PS solution was then transferred into the PEI solution, and this reaction was stirred at 500 rpm at room temperature for 12 h. Next, the B-PEI was precipitated by gently pouring the solution into 1 L acetone, mixing at 500 rpm, and leaving it to mix for 4 h. The excess acetone was decanted, and the precipitate was washed with 500 mL of ethanol and acetone one time for 5 min, to remove unreacted PS via decantation of the supernatant solution. The precipitated viscous gummy B-PEI was placed in an oven at 50 °C to evaporate the acetone, ethanol, and water for 5 h, and the dried B-PEI was stored in a closed container.

The structural analysis of PEI and B-PEI was conducted using FT-IR spectroscopy (Nicolet IS10, Thermo, USA) in the wavenumber range of 650 to 4000 cm^−1^ with a resolution of 4 cm^−1^ using the attenuated total reflectance (ATR) technique.

### 2.3. Protonation of PEI and B-PEI Derivate

PEI and B-PEI were separately protonated with citric acid (CA), boric acid (BA), and hydrochloric acid (HCl) solutions. In brief, 1 wt% of PEI-CA, PEI-BA, and PEI-HCl solutions were separately prepared in 0.1 M CA, 0.1 M BA, and 0.01 M HCl aqueous solutions, respectively. Similarly, B-PEI-CA, B-PEI-BA, and B-PEI-HCl solutions at 1 wt% were separately prepared in 0.1 M CA, 0.1 M BA, and 0.01 M HCl aqueous solutions, respectively. The solution of B-PEI in citric acid (CA), boric acid (BA) and hydrochloric acid (HCl) was placed in a spray bottle.

### 2.4. Cytotoxicity of PEI- and B-PEI-Derived Spray Solutions

The cytotoxicity of PEI- and B-PEI-based solutions was determined using an MTT colorimetric assay in accordance with the literature to assess the cell viability in the presence of PEI-based solutions [34]. L929 fibroblast cells were cultured in DMEM supplemented with 10% (*v*/*v*) FBS and 1% antibiotics as a culture medium at 37 °C, with 5% CO_2_. In brief, 100 μL of a 5 × 10^4^ cell/mL concentration of the cell suspension in culture medium was seeded onto each well in a 96-well plate and incubated for 24 h at 37 °C with 5% CO_2_ to obtain adhesive L929 cells. Then, the culture medium was replaced with 100 μL of different concentrations of PEI-based solution in the range of 50–1000 μg/mL and was added to the adhesive cells. As a control group, the culture medium was replaced with fresh culture medium to obtain untreated cells. The plate was incubated for 24 h at 37 °C, with 5% CO_2_. At the end of the incubation, the PEI-based solution was removed from the wells, and the cells were washed with PBS one time. Separately, 5 mg/mL concentration of MTT reagent was diluted ten times with DMEM, and 100 μL of this reagent was added to each well. The plate was incubated for 2 h in dark conditions, and MTT solution was replaced with 200 μL of DMSO to dissolve the formazan crystals. Then, the absorbance value of the purple color was determined by using a plate reader (Thermo, Multiskan Sky, Waltham, MA, USA) at the 570 nm wavelength. The cell viability % of the cells in the presence of the PEI-based solutions was calculated by means of absorbance of treated cells/absorbance of untreated cells as a control × 100. All assays were performed three times, and the results are given with standard deviations. The statistical analysis was performed using GraphPad Prism 8 software, and the differences between the groups were assessed according to Student’s *t*-test. The results were determined as statistically significant for *p*-values of * *p* < 0.05 and ** *p* < 0.001 vs. control.

### 2.5. Antibacterial and Antifungal Activities of PEI- and B-PEI-Derived Spray Solutions

Microtiter broth dilution and disc diffusion assays were used to determine the antibacterial effect of PEI- and B-PEI-based solutions on Gram-negative *Escherichia coli* (ATCC 8739), *Pseudomonas aeruginosa* (ATCC 10145), and *Klebsiella pneumoniae* (ATCC 700603) and Gram-positive *Staphylococcus aureus* (ATCC 6538) and *Bacillus subtilis* (ATCC 6633) bacterial strains. Similarly, the antifungal activity of PEI- and B-PEI-based solutions was determined against *Candida albicans* (ATCC 10231) and *Mucor* spp. fungal strains using the same procedures.

#### 2.5.1. Microtiter Broth Dilution Assay

In the microtiter broth dilution test, 100 μL of liquid growth medium as the nutrient broth was added to each well in a 96-well plate. Then, 100 μL of 40 mg/mL concentration of PEI solution in DI water was added to the well plate and diluted with the existing liquid growth medium from 20 to 0.15 mg/mL concentrations, respectively. Similarly, gentamicin and amphotericin B were used as control antibiotic and antifungal agents. A bacteria/fungi suspension at 1.5 × 10^8^ CFU/mL of 5 μL was added to each well, and the 96-well plate was incubated at 35 °C overnight for bacteria [35] and at 25 °C for 48 h for fungi. The lowest concentration of PEI solution in a transparent well was determined as the minimum inhibition concentration (MIC) value. Then, 100 μL from all transparent wells was inoculated on nutrient agar, and the lowest concentration of PEI solution with no living bacteria was determined as the minimum bactericidal/fungicidal concentration (MBC/MFC) value.

#### 2.5.2. Disc Diffusion Test

The McFarland 0.5 standard (50 μL of 1% barium chloride suspended in 9.95 mL of 1% sulfuric acid) was used to adjust the bacteria/fungi culture suspension to about 1.5 × 10^8^ CFU/mL, and 0.1 mL of this bacteria/fungi suspension was inoculated on nutrient agar for bacteria or potato dextrose agar for fungal species. Then, 9 mm diameter sterile filter discs were placed on the center of plate, and 50 μL of 10 mg/mL concentration PEI solution in DI water was dropped on the disc. Gentamicin antibiotic at 20 μL with a 1 mg/mL concentration in DI water was used as a control for antibacterial analysis, while amphotericin B (AMB) antifungal drug at 20 μL with a 1 mg/mL concentration in DI water was used as a control for the antifungal activity test. These agar plates were incubated at 35 °C for 18–24 h for antibacterial analysis or left for a 48 h incubation time at 25 °C for fungal growth. The inhibition zone was determined according to the transparent zone diameter (mm) around the filter disc.

### 2.6. Antiviral Activities of PEI- and B-PEI-Derived Spray Solutions

PEI-based polymer was also tested against SARS-CoV-2 virus in the Institute for Antiviral Research, Utah State University in accordance with the accredited procedure. For that purpose, SARS-CoV-2 virus stocks were prepared by growing the virus in Vero 76 cells. PEI-based solutions were prepared at 1% (by weight) in distilled water. SARS-CoV-2 virus stock was added to triplicate tubes of each prepared concentration so that there was 10% virus solution by volume and 90% prepared sample. Medium only was added to one tube of each prepared concentration to serve as toxicity controls. Ethanol was tested in parallel as a positive control and distilled water to serve as the virus control. The prepared compound solutions at 1% (weight/volume in DI water) and virus were incubated at room temperature for 30 min. Following the contact period, the solutions were neutralized by a 1/10 dilution in test media.

The endpoint dilution assay was employed to quantify the surviving virus. Neutralized samples were mixed for quantification to produce an average for triplicate tests. These solutions were serially diluted 10-fold eight times into the medium. Each dilution was placed into the four wells in a 96-well plate containing 80–100% confluent Vero E6 cells. For toxicity control, an extra 4 wells were added, and 2 of these were infected with virus (considered neutralization controls), allowing us to confirm that the remaining sample in the titer assay plate did not inhibit growth and detection of surviving virus. The 96-well plate was incubated for 5 days at 37 ± 2 °C, under 5% CO_2_. At the end of the incubation, post-infection plates were scored for the presence or absence of a viral cytopathic effect (CPE). To uncover the endpoint titers (50% cell culture infectious dose, CCID50) of the sample, the Reed–Muench method was employed. The log reduction value (LRV) of PEI-based materials was calculated and compared with water as a negative control. Virus controls were tested in DI water, where we assessed the reduction in virus in test wells compared to virus controls, calculated as the LRV. To check whether PEI-based material is toxic, media without virus were used against cells. Neutralization controls were tested to make sure that virus deactivation had not proceeded in the stated contact time, and we found that the remaining sample in the titer assay plates did not prevent growth and detection of surviving virus. This investigation was performed by adding toxicity samples to titer test plates and then spiking each well with a lesser amount of virus (about 30 CCID50), which produced an observable amount of CPE during the period of incubation.

### 2.7. Microbial Inhibition Effect of PEI-Based Solution on Mask

The microbial inhibition effects of the PEI-based PEI, PEI-BA, B-PEI, and B-PEI-BA aqueous solutions were tested on a cotton mask against *E. coli*, *P. aeruginosa*, *B. subtilis*, and *S. aureus* as bacteria and *C. albicans* and *Mucor* spp. as fungi [36].

For the inhibition assay, a 10 × 10 mm piece of cotton mask was cut off and placed on the solid agar. Then, 50 µL of PEI-based solution at 1, 5, and 10 mg/mL concentrations were dropped on the mask. After 1 h, 20 µL of 0.5 × 10^8^ CFU/mL bacterial/fungal culture was dropped on the mask previously soaked in the PEI-based solutions. As a negative control, only bacteria/fungal culture was dropped on the bare mask. The plate was incubated at 35 °C in an oven for 24 h for bacterial culture or 25 °C for 48 h for fungal culture. At the end of the incubation, the masks were transferred into 4 mL of serum physiological (SP, 0.9% NaCl aqueous solution) and incubated in the same conditions. After 2 h, the growth of bacteria/fungal colonies was measured by using a plate reader at 590 nm. The cell viability % of microorganisms in the presence of the PEI solutions were calculated by the absorbance of the sample solution/absorbance of negative control × 100. All assays were performed three times, and the results are given with standard deviations.

### 2.8. Microbial Adhesion Test on the Mask Treated with PEI-Based Solutions

Microbial adhesion on the cotton mask treated with 0.1% solutions of PEI, PEI-BA, B-PEI, and B-PEI-BA was tested based on the method proposed by Duan et al., with some modification [37]. PEI solution at a 10 mg/mL concentration equal to 1% was prepared, and 50 μL of this solution was dropped on one piece of cotton mask with a 10 × 10 mm size. After 10 min, the mask was immersed in 1 mL of bacterial or fungal culture, which contained 0.5 × 10^8^ CFU/mL colonies of *E. coli*, *P. aeruginosa*, *S. aureus*, *B. subtilis*, *C. albicans,* or *Mucor* spp., respectively. The mask was incubated for 2 h in the bacterial suspension at 35 °C or fungal suspension at 25 °C. Then, the mask was removed from the bacteria/fungi suspension, excess bacterial/fungal suspension was removed, and the mask was placed in nutrient broth (NB) as a liquid growth medium. The liquid medium containing the mask piece was incubated for 24 h at 35 °C for bacterial species and 48 h at 25 °C for fungal species. At the end of the incubation, the mask was washed with sterile physiological serum (SP, 0.9% NaCl aqueous solution) twice to remove non-attached bacteria/fungi. Next, the washed mask was placed in fresh SP and sonicated for 5 min. The count of attached colonies was measured using a plate reader at 590 nm. As a control group, a bare mask without PEI solution was treated with bacteria/fungi. Inhibition of microbial adhesion % was determined by the absorbance of the PEI-solution-treated mask/absorbance of the bare mask × 100. All assays were performed three times, and the results are given with standard deviations.

## 3. Results

**Figure 1:**
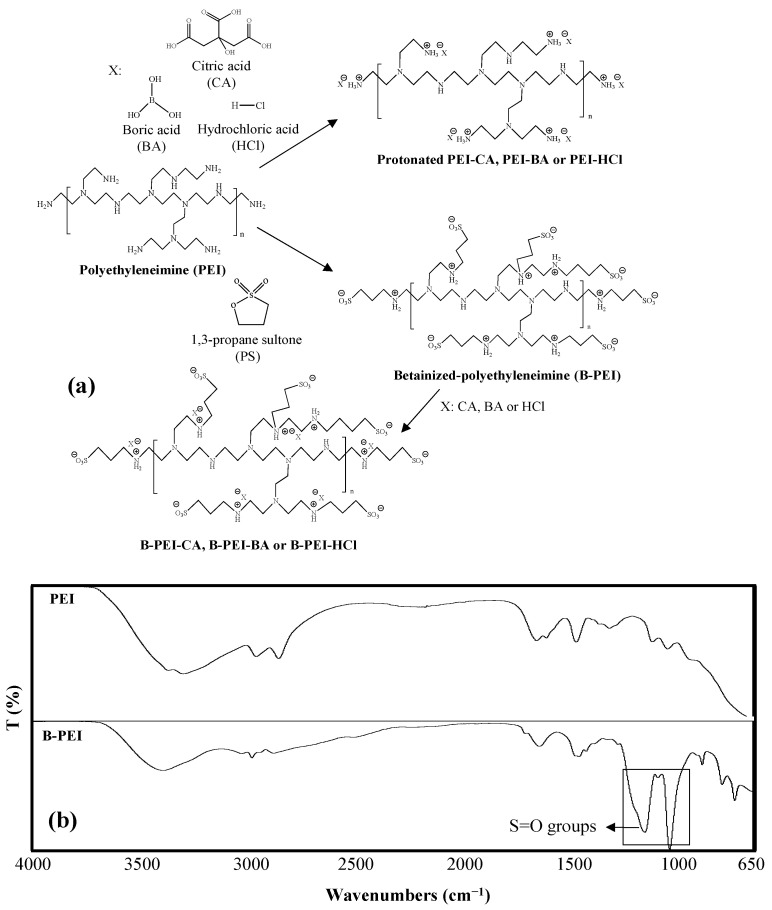
(**a**) Schematic representation of synthesis steps of protonated PEI-CA, PEI-BA, or PEI-HCl solutions, and B-PEI, B-PEI-CA, B-PEI-BA, or B-PEI-HCl solutions. (**b**) FT-IR spectra of PEI and B-PEI.

Different types of protonated PEI solutions of PEI-CA, PEI-BA, and PEI-HCl were prepared separately in a single step by dissolving branched PEI in 0.1 M CA, BA, or 0.01 M HCl aqueous solutions for use as antipathogenic sprays. The synthesis is shown in Figure 1a.

Branched PEI is a well-known antimicrobial agent due to the inherit cationic nature of its primary, secondary, and tertiary amine groups. The antipathogenic activity of PEI can be increased by protonation of the primary amines (mostly) with different acid sources, e.g., CA, BA, or HCl, to generate more cationic groups on PEI chains. In addition, CA, BA, or HCl are separately well-known antiseptic materials and used in a wide range of formulations for cleaning purposes [38,39]. These chemicals can affect different types of microorganisms and could be used to improve the antipathogenic potency of PEI-derived spray. In the spray formulations, the concentrations of acids adopted were 0.1 M for CA and BA and 0.01 M for HCl, providing some antiseptic effects without toxicity. CA and BA are weak acids and afford no significant toxicity as an antiseptic material, but HCl exhibits toxicity due to its strong acid and corrosive nature.

The toxicity of PEI limits its use in antipathogenic spray formulations [26]. To improve the bioactivity of PEI-based spray solutions, PEI was reacted with 1,3-propane sultone (PS). As shown in Figure 1, betainized polyethyleneimine (B-PEI), which also contains SO_3_^−^ groups along with amine groups, was prepared in a single step. The molar ratio of PEI:PS was chosen as 1:1.5 based on PEI repeating units (assuming 44 g/mole). Therefore, each amine group in the polyethyleneimine chain could react with enough 1,3-propane sultone (PS) to source sufficient SO_3_^−^ groups from PS using an excess molar ratio of PS, as some of the amine groups are secondary and tertiary in branched PEI. Then, the B-PEI was dissolved in 0.1 M CA, 0.1 M BA, or 0.01 M HCl aqueous solutions to prepare effective and non-toxic antipathogenic sprays.

The chemical structures of PEI and B-PEI were affirmed via FT-IR analysis, and the corresponding spectra are shown in Figure 1b. Certain peaks of PEI were apparent at 3300 cm^−1^ attributed to N-H vibrations, 1460 and 1350 cm^−1^ for N-H bending, and 1145 and 1045 cm^−1^ belong to C-N stretching vibrations in the spectrum. In addition to the PEI peaks, significant new peaks were observed in the B-PEI spectrum, e.g., at 1032 and 1150 cm^−1^ for stretching vibrations belonging to S=O groups due to the modification of PEI with PS. The presence of these new functional groups on B-PEI confirmed the structural changes in PEI chains upon betainization reaction with PS.

The biological safety of antipathogenic spray is represented by the toxicity level of its ingredients according to the ISO 10993-5 standard [40], which covers the in vitro cytotoxicity of medical or healthcare materials on mammalian cells [40]. In the ISO 10993-5 standard, cell viability levels of ≥100%, 99–75%, 74–50%, 49–25%, and 44–1% represent 0, 1, 2, 3, and 4 toxicity levels, respectively [41]. The toxic effect of the designed PEI-based solutions used as antipathogenic sprays was determined on human primary fibroblast cells upon 24 h incubation.

**Figure 2:**
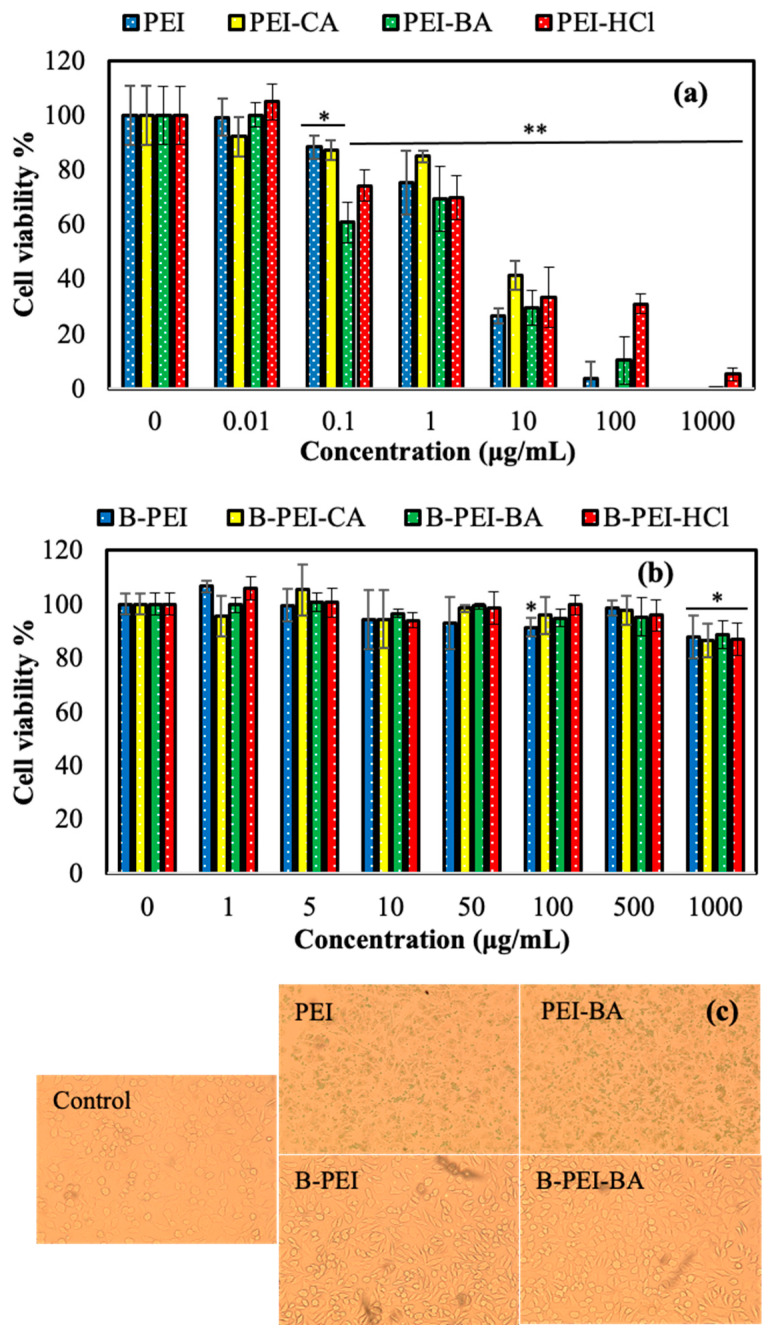
Cytotoxicity of L929 fibroblasts in the presence of (**a**) PEI, PEI-CA, PEI-BA, and PEI-HCl, or (**b**) their betainized forms, B-PEI, B-PEI-CA, B-PEI-BA, and B-PEI-HCl solutions, for a 24 h incubation time up to 1000 μg/mL. (**c**) Light microscope images of the cells interacting with PEI, PEI-BA, B-PEI, and B-PEI-BA solutions at a 1000 μg/mL concentration and without any materials as the control group. The results were determined as statistically significant for *p*-values of * *p* < 0.05 and ** *p* < 0.001 vs. control.

As shown in Figure 2a, toxicity of the branched PEI and its protonated forms at a 1 μg/mL concentration had a relatively acceptable level of 1–2 with 75 ± 11, 85 ± 2, 69 ± 1, and 70 ± 8 % cell viability values for PEI, PEI-CA, PEI-BA, and PEI-HCl solutions, respectively. The cell viability % of L929 fibroblasts was significantly decreased for 10 μg/mL PEI-based solutions with below 40% cell viability values, which resulted in level 4 toxicity. Betainization of PEI with 1,3-propane sultone surpassed the toxicity of the PEI solution [33]. The B-PEI form could overcome the limitations of PEI solution as an antipathogenic spray linked to high toxicity. As demonstrated in Figure 2b, no significant toxicity was determined on the fibroblasts in the presence of a 500 μg/mL concentration of B-PEI and its protonated forms compared with the control group. Furthermore, level 1 toxicity was found even at a 1000 μg/mL concentration of B-PEI, B-PEI-CA, B-PEI-BA, and B-PEI-HCl, with 88 ± 8, 86 ± 6, 89 ± 5, and 87 ± 6% cell viability values, respectively. Light microscope images of the cells interacting with PEI, PEI-BA, B-PEI, and B-PEI-BA solutions at a 1000 μg/mL concentration and in the control group are given in Figure 2c. It can clearly be seen that the cell structure of the fibroblast cells was destroyed by PEI and PEI-BA but was healthy in the presence of B-PEI and B-PEI-BA forms. PEI-based solutions as antipathogenic sprays could be acceptable with a safety level up to a 1 μg/mL concentration, while B-PEI and its protonated forms such as B-PEI-CA, B-PEI-BA, and B-PEI-HCl exhibited no significant toxicity even at a 1000 μg/mL concentration and can thus be safely used as antipathogenic sprays.

Antimicrobial activities of PEI-based antipathogenic sprays were compared for a wide range of microorganisms including *E. coli*, *K. pneumoniae*, and *P. aeruginosa* as Gram-negative bacteria, *S. aureus* and *B. subtilis* as Gram-positive bacteria, and *C. albicans* and *Mucor* spp. as fungal strains. Minimum inhibition concentration (MIC) and minimum bactericidal concentration (MBC) values for different formulations of the PEI sprays on Gram-negative bacteria are given in Table 1.

As the results revealed, PEI solution has low MIC values, e.g., 0.62, 1.25, and 2.5 mg/mL against *E. coli*, *K. pneumoniae*, and *P. aeruginosa*, respectively. Moreover, total bacterial eradication was achieved at 1.25, 2.5, and 2.5 mg/mL MBC values against *E. coli*, *K. pneumoniae*, and *P. aeruginosa*, respectively. The antibacterial effect of PEI solution was significantly enhanced by protonation with BA and HCl, which resulted in 0.62 and 0.31 mg/mL MBC values for both Gram-negative bacteria, but antibacterial activity was only slightly increased for CA protonation of PEI solution, with a 1.25 mg/mL MBC value for each bacterium. Antibacterial potency of betainized PEI solution decreased further because of improved biocompatibility. As can be seen in Table 1, B-PEI had 5 mg/mL MIC values and 20 mg/mL MBC values for Gram-negative bacteria. After the protonation with CA of B-PEI, the antibacterial potency was not significantly changed, but the antibacterial effectiveness of B-PEI-BA and B-PEI-HCl solutions was two-fold increased, with 2.5, 5.0, and 2.5 mg/mL MIC values for B-PEI-BA and 2.5, 2.5, and 1.25 mg/mL MIC values for B-PEI-HCl against *E. coli*, *K. pneumoniae*, and *P. aeruginosa*, respectively.

Antibacterial effects of the prepared PEI-based spray solutions on Gram-positive bacteria of *S. aureus* and *B. subtilis* are listed with MIC and MBC values in Table 2.

According to the results, PEI had 1.25 mg/mL MIC and 2.5 mg/mL MBC values against *S. aureus* and 0.62 mg/mL MIC and 1.25 mg/mL MBC values against *B. subtilis*. This antibacterial effect was increased by protonation of PEI with CA, BA, and HCl, and the highest antibacterial capacity was determined for PEI-BA and PEI-HCl formulations, with 0.62 and 0.31 mg/mL MBC values against *S. aureus* and *B. subtilis*, respectively. Similar to Gram-negative bacteria, the antibacterial effect of PEI was slightly decreased by a betainized PEI (B-PEI) solution for Gram-positive bacteria, with 2.5 and 1.25 mg/mL MIC values against *S. aureus* and *B. subtilis*, respectively. The highest antibacterial property was observed for the B-PEI-BA formulation against *S. aureus* and *B. subtilis*, with a two-fold lower MBC value than B-PEI.

The antifungal activity of PEI-based formulations was also investigated against *C. albicans* and *Mucor* spp., as given in Table 3.

The MIC and MFC values of PEI were determined as 0.31 and 0.62 mg/mL for both fungi. The antifungal effect was nearly two-fold increased by protonation of PEI with CA, BA, and HCl, and the best antifungal activity was observed for the PEI-BA formulation, with 0.31 and 0.15 mg/mL MFC values against *C. albicans* and *Mucor* spp., respectively. For the B-PEI-based spray solutions, the B-PEI-BA formulation exhibited the highest antifungal activity, with 2.5 and 5 mg/mL MIC values against *C. albicans* and *Mucor* spp., respectively. These results imply that protonation of PEI or B-PEI with BA or HCl significantly improved the antimicrobial activity of these compounds. In particular, BA protonated with PEI or B-PEI had the greatest broad-spectrum antipathogenic effect against Gram-negative and Gram-positive bacteria as well as fungal species.

Therefore, the antimicrobial activities of PEI, PEI-BA, B-PEI, and B-PEI-BA formulations at a 10 mg/mL concentration in 50 μL were also investigated by a disc diffusion assay against various Gram-negative and Gram-positive bacteria and fungal species.

**Figure 3:**
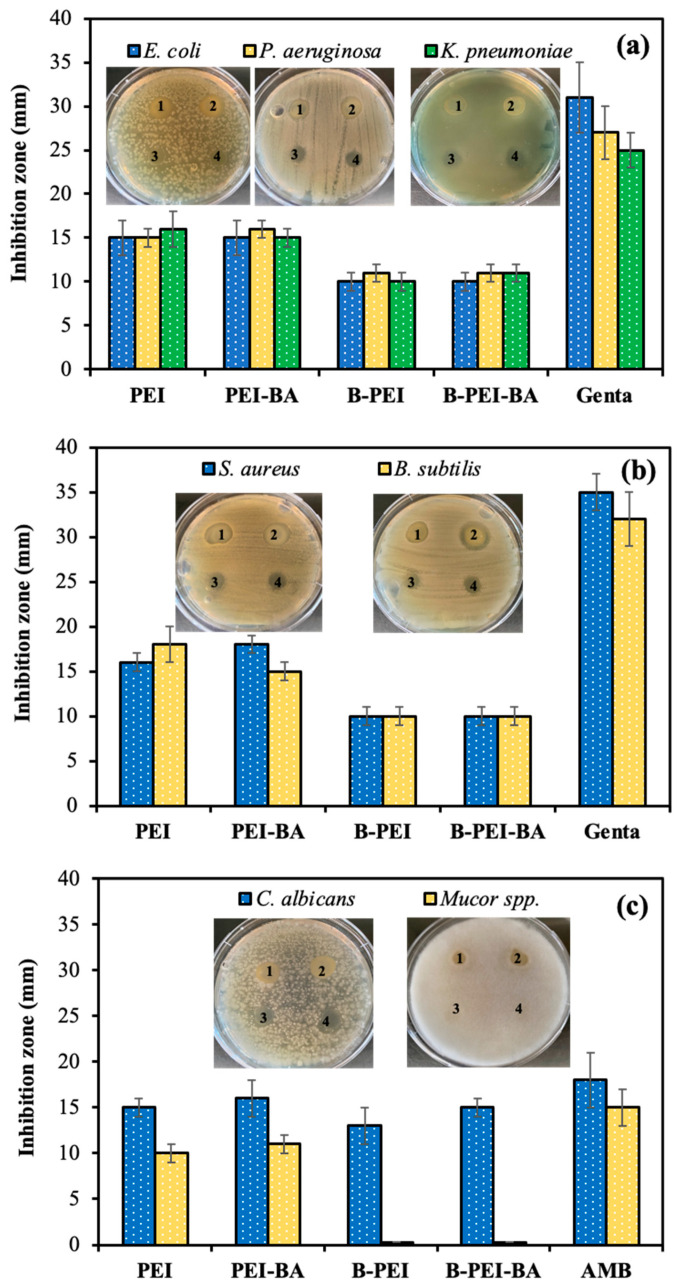
Inhibition zone diameter (mm) of (1) PEI, (2) PEI-BA, (3) B-PEI, and (4) B-PEI-BA at 50 μL of 10 mg/mL concentration against (**a**) *E. coli*, *P. aeruginosa*, and *K. pneumoniae* as Gram-negative bacteria, (**b**) *S. aureus* and *B. subtilis* as Gram-positive bacteria, and (**c**) *C. albicans* and *Mucor* spp. as fungal strains.

As can be seen in Figure 3a, the inhibition zone of PEI was slightly increased for the PEI-BA formulation from 15 ± 2 mm to 16 ± 2 mm against Gram-negative bacteria. Furthermore, the 10 ± 1 mm zone diameter of B-PEI was also increased to 11 ± 1 mm for the B-PEI-BA spray against *K. pneumoniae*. As shown in Figure 3b, the antibacterial activity of PEI with a 15 ± 2 mm inhibition zone was increased to 18 ± 2 mm for the PEI-BA formulation but did not change for the B-PEI and B-PEI-BA solutions with a 10 ± 1 mm inhibition zone against Gram-positive *S. aureus* and *B. subtilis*. In addition, the antifungal effects of PEI and PEI-BA were 15 ± 1 and 16 ± 2 mm against *C. albicans*, respectively. The antifungal activity of B-PEI was slightly decreased to 13 ± 2 mm, but the inhibition efficacy was measured as 15 ± 1 mm for the B-PEI-BA spray against *C. albicans*. For the other harmful pathogen *Mucor* spp., the inhibition zones were 10 ± 1 and 11 ± 1 mm for PEI and PEI-BA, but no inhibition was seen for the B-PEI and B-PEI-BA forms. As revealed by the results, microbial inhibition could be improved by using protonated PEI-BA formulations. Moreover, the effectiveness was slightly decreased by B-PEI because of its improved biocompatibility, while the antimicrobial activity of B-PEI-BA was similar to that of PEI, except for *Mucor* spp.

Antiviral activities of PEI formulations against SARS-CoV-2 virus were determined as mentioned in Section 2.6. The virus titers and log reduction values (LRVs) for samples tested against SARS-CoV-2 are shown in Table 4.

Some of the compounds given Table 4 also had antiviral activities. As can be seen, bare (unmodified) PEI can reduce the virus by 90% (LRV 1.0). Moreover, PEI-HCl (HCl-treated/modified PEI) and PEI-CA (citric-acid-treated/modified PEI) can reduce the virus > 90% (LRV > 1.6) at the studied concentration, 1%.

The virus control titer, DI water, was used at 4.3 log CCID50 per 0.1 mL for comparisons of all test sample titers to determine the LRV. Samples with <1 log reduction were not considered active for virucidal activity.

The limit of detection of virus for samples that did not exhibit cytotoxicity when plated for the endpoint dilution assay was 0.7 log CCID50 per 0.1 mL. When >80% cytotoxicity was observed in wells of diluted samples, the presence of a virus could not be ruled out and therefore the limit of detection was altered. For instance, when cytotoxicity was seen in the 1/10 dilution, the limit of detection was 1.7 logs, in 1/100, it was 2.7 logs, and so forth.

As can be seen from Table 5, the betainized forms of PEI (B-PEI, B-PEI-HCl, B-PEI-BA) exhibited virucidal activity, reducing the SARS-CoV-2 titer by more than 3 log CCID50 per 0.1 mL (>99.9%).

It is apparent that betainized forms of PEI, B-PEI, B-PEI-HCl, B-PEI-BA, and B-PEI-CA solutions are very effective against SARS-CoV-2 and can be safely used in real applications without any concerns as the biocompatibility is significantly improved upon betainization.

The use of these PEI-based formulations as antipathogenic sprays was demonstrated by the inhibition of microbial viability on a face mask in the presence of different concentrations of PEI-based solutions, from 1 to 10 mg/mL concentrations, which contain 0.1%, 0.5%, and 1 wt% of PEI-based materials.

**Figure 4:**
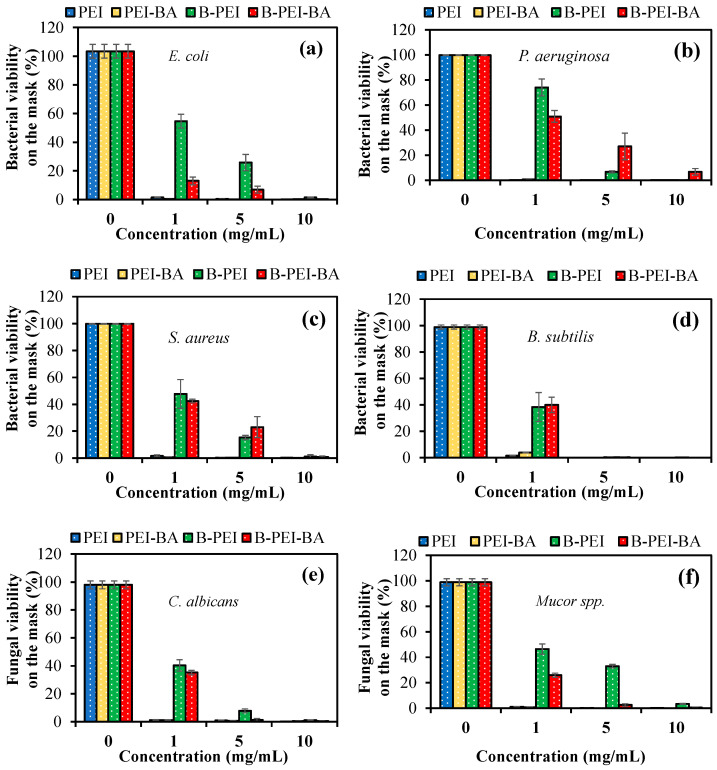
Inhibition of bacterial cell viability% of (**a**) *E. coli*, (**b**) *P. aeruginosa*, (**c**) *S. aureus*, (**d**) *B. subtilis*, (**e**) *C. albicans*, and (**f**) *Mucor* spp. on a mask in the presence of 50 μL of 1, 5, and 10 mg/mL of PEI-based solution.

As illustrated in Figure 4, PEI and PEI-BA sprays totally killed all types of microorganisms, even at the 1 mg/mL concentration, which equals a 0.1% spray solution. MIC values of the PEI and PEI-BA solutions were generally below 1 mg/mL, and one drop of 50 μL of 0.1% PEI or PEI-BA spray solution was enough to eradicate the bacterial and fungal species. Microbial viability% results on the mask in the presence of B-PEI spray solution at a 1 mg/mL concentration were 54 ± 5, 74 ± 7, 47 ± 10, 38 ± 10, 40 ± 4, and 46 ± 1% against *E. coli*, *P. aeruginosa*, *S. aureus*, *B. subtilis*, *C. albicans,* and *Mucor* spp., respectively. The same concentration of B-PEI-BA spray solution could remove more than half of the microbial colony on the mask, with values of 13 ± 2, 50 ± 4, 42 ± 1, 40 ± 5, 35 ± 1, and 25 ± 5% against *E. coli*, *P. aeruginosa*, *S. aureus*, *B. subtilis*, *C. albicans,* and *Mucor* spp., respectively. Furthermore, 50 μL of 0.5% B-PEI-BA spray could inhibit almost all bacterial and fungal species on the mask. These results lead us to assert that 50 μL of 0.5% B-PEI and B-PEI-BA solutions could be used as antipathogenic sprays due to their great microbial inhibition ability in addition to low toxicity.

**Figure 5:**
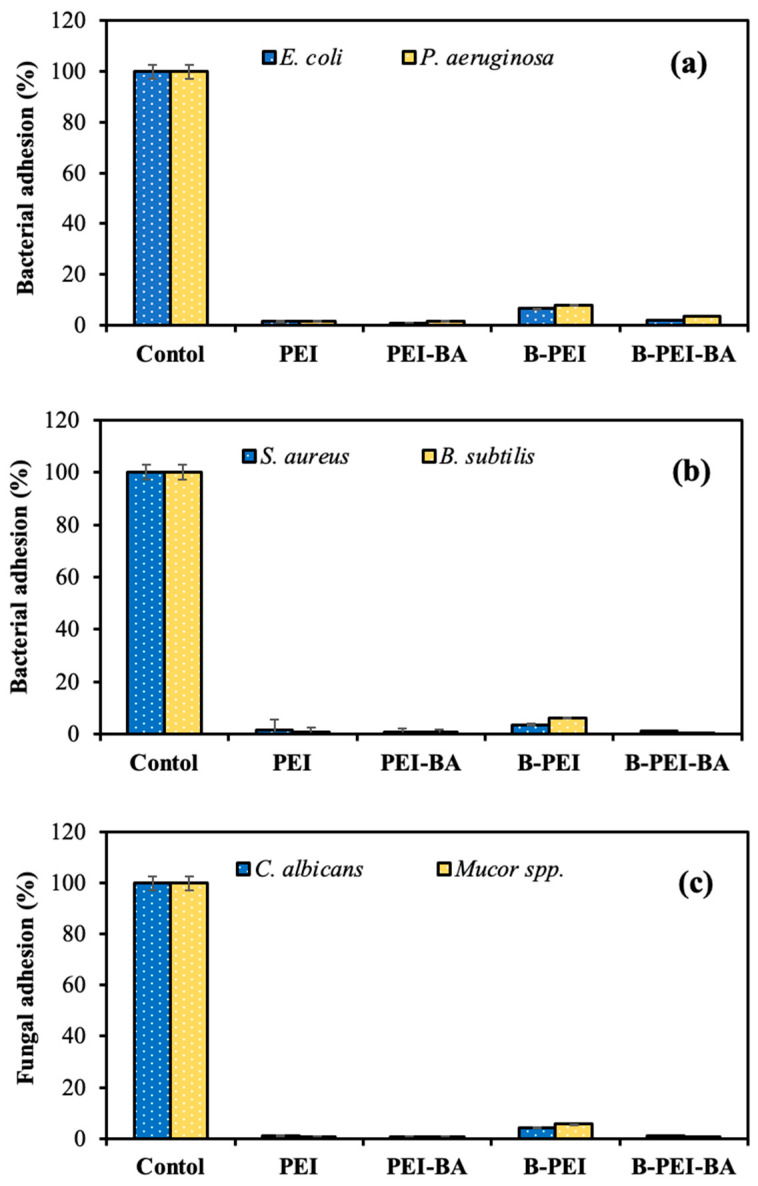
Inhibition of bacterial/fungal adhesion% of (**a**) *E. coli*, *P. aeruginosa*, (**b**) *S. aureus*, *B. subtilis*, and (**c**) *C. albicans*, *Mucor* spp. on a mask in the presence of 50 μL of 10 mg/mL of PEI-based solution.

Inhibition of bacterial or fungal adhesion on the mask by 50 μL of 1% concentration of PEI-based spray solution was investigated by comparison with the control group, which was the bare mask, and the results are given in Figure 5.

As can be seen in Figure 5a–c, PEI and PEI-BA sprays provided more than 98% inhibition of bacterial/fungal adhesion of Gram-negative and Gram-positive bacteria and fungal species. In addition, microbial adhesion% results for the face mask with B-PEI spray were 6.5 ± 0.4, 8.1 ± 4.2, 3.6 ± 0.2, 6.1 ± 2.3, 4.2 ± 1.5, and 5.5 ± 2.1 for *E. coli*, *P. aeruginosa*, *S. aureus*, *B. subtilis*, *C. albicans,* and *Mucor* spp., respectively. But these adhered bacteria or fungi were almost totally eradicated by using B-PEI-BA as an antipathogenic spray, similar to PEI and PEI-BA solutions.

## 4. Discussion

The common antipathogenic solutions of alcohol-based sanitizers cause protein denaturation and destroy the lipid membrane through dissolving and dehydration effects on microorganisms [10]. Andal et al. reported that alcohol could interact with the lipid layer of the SARS-CoV-2 virus and rupture the weak non-covalent interactions between protein and lipid bilayer, in addition to dissolving the lipids in the viruses [42]. Other alcohol-free sanitizers consist of chlorhexidine, quaternary ammonium, triclosan, iodine, hydrogen peroxide, and benzalkonium chloride, which are effective antiseptic chemicals [10]. These are generally cheap and easily produced antipathogenic ingredients, but low biosafety with a high toxicity level for humans and narrow microbial activity [10] has led to the design of novel antipathogenic products. Recently, metal oxides, metal nanoparticles, quantum dots, carbon dots, cationic polymeric structures, carbon-based materials, and so on were also considered as antipathogenic materials due to their ability to cause membrane damage, their ROS generation ability, and their genetic material affecting the microorganism. As an alternative antiviral material, copper cold spray was prepared, and the antiviral mechanism of this product was explained as ROS generation or genomic/membrane damage to the viruses by the spray [9]. Similar to these well-known antiseptic chemicals or advanced materials, the microbiocidal mechanism of PEI was explained by damage to the membrane structure, genetic materials, or certain enzymes through the higher bonding affinity of cationic PEI chains, in addition to destruction of the protein, lipid, or DNA structure by ROS production in pathogenic organisms. These different killing effects of PEI provide broad-spectrum antibacterial, antifungal, or antiviral activities, which were supported by our results with low MBC and MFC values in addition to high zone diameters against common hospital pathogens such as *E. coli*, *P. aeruginosa*, and *K. pneumoniae* as Gram-negative bacteria, *S. aureus* and *B. subtilis* as Gram-positive bacteria, *C. albicans* and *Mucor* spp. as fungi, and the SARS-CoV-2 virus.

In the literature, there are some results about the antimicrobial activity of branched PEI for a wide range of microorganisms [26,43,44], but the design and incorporation of betaine, e.g., B-PEI formulations as antipathogenic sprays, was investigated for the first time in this study. To use PEI in biomedical applications, some modification or complexation with different natural and synthetic molecules such as cellulose [45], hyaluronic acid [46], polyphenolic compounds [47], and so on is required because of its toxicity limitation. These formulations unweave the toxicity and allow significant antimicrobial activity. Similarly, our results revealed that PEI and its protonated forms of PEI-CA, PEI-BA, and PEI-HCl possess level 1 toxicity at the 1 μg/mL concentration, but no biosafety at the 10 μg/mL concentration according to the ISO 10993-5 standard, which is an acceptable procedure to use to detect the cytotoxicity of healthcare products such as antipathogenic sprays [40]. As reported by Sahiner et al., cell toxicity of PEI could be managed by a simple betainization reaction with 1,3-propane sultone [33]. Our results indicate that toxicity of the more cationic PEI solution can be overcome by using B-PEI forms with great cell viability for fibroblasts even at a 1000 μg/mL concentration and its protonated forms of B-PEI-CA, B-PEI-BA, and B-PEI-HCl.

It is well-known that branched PEI is a strong antibacterial and antifungal molecule with a polyamine nature. Lan et al. reported the antibacterial activity of PEI and quaternized ammonium PEI prepared with different average molecular weights (M_n_) of PEI from 600 to 70,000. Low M_n_ of PEI at 1200–1800 had the highest antibacterial effect, and approximately a 0.5 mg/mL concentration of PEI could eradicate the biofilm layer of pathogenic bacteria, affecting more than 80% of the biofilm [26]. In our previous study, the MBC/MFC values of PEI for Mn: 1800 were 0.5–1 mg/mL against Gram-negative and Gram-positive bacteria and *Candida albicans* fungus [44]. In this study, PEI sprays were prepared with M_n_: 60,000 of PEI, and the antibacterial and antifungal results were supported by the literature, with 0.62–2.5 mg/mL MBC/MFC values reported for the same bacterial and fungal species. It could be said that the molecular weight value affected the antipathogenic activity of PEI and that the bacteriocidic effect of our formulations could be improved by using a low M_W_ of PEI.

Citric acid (CA), boric acid (BA), and HCl are well-known antiseptic molecules, and 1–3% concentrations of these acids are commonly used for infection removal [38,39]. PEI solution was separately dissolved in 0.1 M CA, 0.1 M BA, or 0.01 M HCl solutions to improve the antipathogenic activity of PEI. Antibacterial and antifungal effects of PEI-CA were not significantly changed, but the MBC/MFC value of PEI-BA and PEI-HCl sprays was nearly 4-fold decreased with more antipathogenic effect than PEI. In addition, antimicrobial activity was significantly reduced in the non-toxic B-PEI formulation when compared with PEI, but the activity was improved by protonation of B-PEI with BA or HCl. These results revealed that PEI or B-PEI solutions can have a synergistic antimicrobial effect via a protonation reaction with different acids such as BA, CA, and HCl. In the mask experiments, the antipathogenic effects of PEI, PEI-BA, B-PEI, and B-PEI-BA were studied and applied to a mask at 50 μL with a 1–10 mg/mL concentration range, equal to 0.1–1 wt% active ingredient. Approximately 1% of B-PEI and B-PEI-BA totally killed all types of colonies and eradicated the adhesion of the pathogens. Gibney et al. reported that interactions of PEI with the outer membrane of *E. coli* gradually increased for 1 h and permeability ended within 1.5 h [43]. The persistence times of *E. coli*, *P. aeruginosa*, *Klebsiella* spp., and *Staphylococcus aureus* bacteria on dry inanimate surfaces were reported to be 1.5 h–16 months, 6 h–16 months, 2 h–30 months, and 7 days–7 months, respectively. In addition, *C. albicans* could survive on a dry surface from 1 to 120 days, but virus survival was found to be only 3 h for coronavirus [7]. These studies show that our prepared PEI formulations could interact with the membranes of the microorganism and penetrate them quickly to remove the microorganisms from the surfaces. These PEI-based formulations can be utilized as antipathogenic sprays, not only for the elimination of microorganisms but also for protecting material surfaces against hazardous microbes or keeping the surfaces sterile for extended time periods, which the commercially available sprays cannot. Moreover, these types of spray solutions can be readily used in public places, on medical equipment, textile products, or healthcare products, in household applications, in animal care products, and so on with long lifetimes and without harming the environment.

## 5. Conclusions

To design alternative antipathogenic spray solutions, PEI and B-PEI solutions and their protonated forms with CA, BA, and HCl were prepared and tested for antipathogenic properties. Branched PEI and its protonated forms exhibited low toxicity on fibroblast cells up to a 1 μg/mL concentration, with 75 ± 11% cell viability, but significant toxic effects at >10 μg/mL concentration were observed. To avoid toxicity, PEI was betainized with 1,3-propane sultone, and the cell toxicity of PEI solutions significantly decreased for B-PEI and its protonated forms, with level 1 toxicity up to a 1000 μg/mL concentration accomplished. The excellent antibacterial/fungal effects of PEI were significantly decreased in the B-PEI form, but the antimicrobial activity improved again with protonation of B-PEI with BA and HCl. A broad-spectrum antimicrobial capability was obtained for each formulation against harmful bacteria and fungi. Combining B-PEI with HCl, BA, and CA produced protonated forms that showed very potent antiviral properties, especially against SARS-CoV-2 virus. In the administration of PEI spray solutions on a mask, no bacterial and fungal viability was determined at a 10 mg/mL concentration for non-toxic B-PEI and B-PEI-BA sprays, as well as no microbial adhesion. Thus, B-PEI-BA can be recommended as an alternative antipathogenic spray given its simple preparation process, good biocompatibility, and inhibition of microbial growth and microbes’ ability to adhere to masks and other surfaces, along with improved antiviral properties. Therefore, we conclude that the B-PEI-BA spray formulation has great potential as an antipathogenic spray with not only broad-spectrum antimicrobial effectiveness but also high antiviral properties and excellent biocompatibility.

## Figures and Tables

**Table 1:** Minimum inhibitory concentration (MIC) and minimum bactericidal concentration (MBC) values of PEI-based solutions against *E. coli* (ATCC 8739), *K. pneumoniae* (ATCC 700603), and *P. aeruginosa* (ATCC 10145) as Gram-negative bacteria.

Sample	*E. coli*		*P. aeruginosa*		*K. pneumoniae*	
	MIC (mg/mL)	MBC (mg/mL)	MIC (mg/mL)	MBC (mg/mL)	MIC (mg/mL)	MBC (mg/mL)
**PEI**	0.62	1.25	1.25	2.5	2.5	2.5
**PEI-CA**	0.62	1.25	0.62	1.25	0.62	1.25
**PEI-BA**	0.31	0.62	0.62	0.62	0.62	0.62
**PEI-HCl**	0.31	0.31	0.31	0.31	0.31	0.31
**B-PEI**	5.0	20.0	5.0	20.0	5.0	10.0
**B-PEI-CA**	5.0	20.0	10.0	20.0	5.0	10.0
**B-PEI-BA**	2.5	10.0	5.0	10.0	2.5	10.0
**B-PEI-HCl**	2.5	10.0	2.5	5.0	1.25	10.0
**Gentamicin**	0.002	0.06	0.004	0.06	0.0009	0.008

**Table 2:** Minimum inhibitory concentration (MIC) and minimum bactericidal concentration (MBC) values of PEI-based solutions against *S. aureus* (ATCC 6538) and *B. subtilis* (ATCC 6633) as Gram-positive bacteria.

Samples	*S. aureus*		*B. subtilis*	
	MIC (mg/mL)	MBC (mg/mL)	MIC (mg/mL)	MBC (mg/mL)
**PEI**	1.25	2.5	0.62	1.25
**PEI-CA**	0.62	0.62	0.62	0.62
**PEI-BA**	0.62	0.62	0.31	0.31
**PEI-HCl**	0.62	0.62	0.31	0.31
**B-PEI**	2.5	10.0	1.25	5.0
**B-PEI-CA**	2.5	10.0	1.25	5.0
**B-PEI-BA**	2.5	5.0	0.62	2.5
**B-PEI-HCl**	2.5	10.0	0.62	1.25
**Gentamicin**	0.009	0.1	0.0004	0.1

**Table 3:** Minimum inhibitory concentration (MIC) and minimum fungicidal concentration (MFC) values of PEI-based solutions against *C. albicans* (ATCC 10231) and *Mucor* spp. as fungal strains.

Sample	*C. albicans*		*Mucor* spp.	
	MIC (mg/mL)	MFC (mg/mL)	MIC (mg/mL)	MFC (mg/mL)
**PEI**	0.31	0.62	0.31	0.62
**PEI-CA**	0.15	0.31	0.15	0.31
**PEI-BA**	0.15	0.31	0.15	0.15
**PEI-HCl**	0.15	0.31	0.15	0.31
**B-PEI**	5.0	20.0	10.0	20.0
**B-PEI-CA**	5.0	20.0	2.5	5.0
**B-PEI-BA**	2.5	10.0	5.0	5.0
**B-PEI-HCl**	2.5	10.0	10.0	10.0

**Table 4:** The antiviral activities of PEI-based compounds against SARS-CoV-2 at 30 min incubation at 22 ± 2 °C.

Compound	Concentration	Contact Time (min)	Toxicity ^a^	Neut. Control ^b^	Virus Titer ^c^	VC Titer ^c^	LRV ^d^
**DI Water**	100%	30	None	None	4.3	4.3	0
**PEI**	100%	30	1/100	None	3.3	4.3	1.0
**PEI-HCl**	100%	30	1/100	None	<2.7	4.3	>1.6
**PEI-BA**	100%	30	1/100	None	4.3	4.3	0
**PEI-CA**	100%	30	1/100	None	<2.7	4.3	>1.6
**Ethanol**	70%	30	None	None	<0.7	4.3	>3.6

^a^ Cytotoxicity indicates the highest dilution of the endpoint titer where full (80–100%) cytotoxicity was observed. ^b^ Neutralization control indicates the highest dilution of the endpoint titer where the compound inhibited virus CPE in the wells after neutralization (ignored for calculation of virus titer and LRV). ^c^ Virus titer of test sample or virus control (VC) in log10 CCID50 of virus per 0.1 mL. ^d^ LRV (log reduction value) is the reduction in virus in the test sample compared to the virus control.

**Table 5:** B-PEI-based compounds that show virucidal activity against SARS-CoV-2 after 30 min incubation at 22 ± 2 °C.

Compound	Concentration	Contact Time (min)	Toxicity ^a^	Neut. Control ^b^	Virus Titer ^c^	VC Titer ^c^	LRV ^d^
**B-PEI**	100%	30	None	None	0.7	4.3	3.6
**B-PEI-HCl**	100%	30	None	None	<0.7	4.3	>3.6
**B-PEI-BA**	100%	30	None	None	<0.7	4.3	>3.6
**B-PEI-CA**	100%	30	None	None	4.0	4.3	0.3

^a^ Cytotoxicity indicates the highest dilution of the endpoint titer where full (80–100%) cytotoxicity was observed. ^b^ Neutralization control indicates the highest dilution of the endpoint titer where the compound inhibited virus CPE in the wells after neutralization (ignored for calculation of virus titer and LRV). ^c^ Virus titer of test sample or virus control (VC) in log10 CCID50 of virus per 0.1 mL. ^d^ LRV (log reduction value) is the reduction in virus in the test sample compared to the virus control.

## Data Availability

Data is contained within this article.
